# Incidence and predictors of loss to follow up among HIV-infected adults at Pawi General Hospital, northwest Ethiopia: competing risk regression model

**DOI:** 10.1186/s13104-018-3407-5

**Published:** 2018-05-10

**Authors:** Moges Agazhe Assemie, Kindie Fentahun Muchie, Tadesse Awoke Ayele

**Affiliations:** 1 Department of Public Health, College of Health Sciences, Debre Markos University, Debre Markos, Ethiopia; 2 Department of Epidemiology and Biostatistics, Institute of Public Health, College of Medicine and Health Sciences, University of Gondar, Gondar, Ethiopia

**Keywords:** Lost to follow-up, Cumulative incidence, Competing risks regression, Sub-distribution model, Predictors, Associated factors, ART

## Abstract

**Objective:**

This study was aimed at assessing the incidence of lost-to-follow-up and its predictors among HIV-positive adults after initiation into antiretroviral therapy at Pawi General Hospital, northwest Ethiopia.

**Results:**

The overall cumulative incidence of lost-to-follow-up after ART initiation was high, 11.6 (95% CI 9.8–13.7) per 100 adult-years follow-up time. Independent significant predictors of lost to follow up were being aged 15–28 years (aSHR = 0.44; 95% CI 0.24–0.83), being on WHO clinical stage IV (aSHR = 2.09; 95% CI 1.02–3.13); and receiving isoniazid preventive therapy (aSHR = 0.11; 95% CI 0.06–0.18).

**Electronic supplementary material:**

The online version of this article (10.1186/s13104-018-3407-5) contains supplementary material, which is available to authorized users.

## Introduction

Globally, 17 million people were accessing antiretroviral therapy (ART), coverage being 46% at the end of 2015 [1]. In Ethiopia, nearly 1.2 million people were living with HIV/AIDS with an incidence of 1.2% [2]. At the end of 2013, the proportion of patients who ever started ART was 60.7%, of whom 18.7% were lost-to-follow-up (LTFU) in Ethiopia [3].

Although ART significantly reduced mortality and improved life expectancy of HIV-infected patients, the attainment depends on regular patient follow-up [4]. Effective ART can control the virus and prevent transmission. A trial in 2011 showed that adherence to an effective ART reduced the risk of transmitting the virus to their uninfected sexual partner by 96% [5]. LTFU, undesirably blocks the immunological benefits of ART and increases AIDS-related hospitalizations, morbidity, and death in resource limited countries, has emerged as an everyday threat [6, 7]. The incidence of LTFU was estimated to be 10.9 per 100 person-years in South Africa [8], 10 per 100 in Zambia [9], and 8.2 per 100 in Aksum-northern Ethiopia [10].

The significant risk factors of LTFU are sex, youth, occupation, and lack of formal education [11–13] as well clinical characteristics, like CD4-count, WHO stage, functional status and Isoniazid Preventive Therapy (IPT) [9, 12, 14–16].

The analysis of time-to-LTFU can be complicated by competing risks, deaths that alter the probability or completely preclude the occurrence of LTFU. This is distinct from censoring which merely prevents observing the time at which LTFU occurs [17–19]. In standard Kaplan–Meier analyses, those experiencing a competing event will be taken as censored. However, this is invalid because LTFU can no longer occur in those experiencing death, and such analyses will therefore overestimate the probability of the LTFU. Consequently, the method of Fine and Gray, competing-risks regression provides a useful alternative to Cox regression for time to event data in the presence of competing risks [18, 20, 21].

There was limited evidence on the incidence of LTFU and its predictors in the study area. This study aimed to assess the incidence of LTFU and its predictors with death as a competing event among adult HIV positive patients who started ART at Pawi General Hospital, northwest Ethiopia.

## Main text

### Methods

An institution-based retrospective study was conducted at Pawi General Hospital. Pawi General Hospital is found in Pawi town of Benshangul Gumuz Regional State, northwest Ethiopia. The hospital has been providing chronic HIV care and support to both pre-ART and ART clients since 2005. Out of the 3210 patients enrolled for the care, 1760 had started ART in the hospital.

All HIV-infected adults who enrolled at Pawi General Hospital ART clinic in January 2012–December 2016 were considered for this study. To check sufficiency of samples, minimum sample size (583) was determined for the incidence and predictors using st power command of software Stata 14. Those who had at-least one follow-up visit were eligible to be included. We excluded those who had unknown initiation date, undefined outcome, and transferred in with incomplete base-line data minimizing important factors.

The data were collected using a standard data extraction sheet, entered using Epi Info version 7, and exported to statistical packages Stata version 14.0 for further analysis. The outcome variable in this study was time to LTFU (in months) with death as a competing event. Accordingly, participants were classified as either LTFU, died, or censored (transferred out or still under follow-up) at the end of the study period. ‘LTFU’ is defined as patients not taking ART refill for a period of 3 months or longer from the last attendance for refill and not yet classified as ‘died’ or ‘transferred-out’ [22]. ‘Transferred out’ are patients who were formally transferred out to another health facility. Besides, those patients who died and confirmed by physicians with the approval of the medical director of the hospital followed by recoded final outcome as death on the follow up chart were taken as died. Furthermore, a patient was taken as working functional status if a patient is able to perform usual work in or out of the house.

Incidence of LTFU was determined from the start of ART until the last follow-up visit or known date of death. We used competing risks regression with proportional sub-distribution hazard model to model cumulative incidence [23, 24]. The model quantifies the instantaneous risk of failure from the LTFU and the overall impact of covariates on the incidence of LTFU [17], treating death as a competing risk [18, 23].

Variables in the bi-variable proportional sub-distribution hazard model with p value below 0.25 were subsequently included in the multivariable analysis. Schoenfeld residual and Cox Snell residual plot for the overall model fitness tests were used to check the model assumption. Results were taken as statistically significant for p < 0.05. Crude sub-hazard ratio (cSHR) and adjusted sub-hazard ratio (aSHR) with their respective 95% confidence interval (CI) were reported to show the strength of association.

### Results

Six hundred two HIV infected adults were included in the final analysis. Of these, 4.2% died and 22.6% LTFU. More than half, 338 (56.2%), of HIV positive adults were females. The median age at ART initiation was 32 years [IQR: 27–39]. About 189 (31.4%) of the participants were aged 1528 years, whereas nearly half, 285 (47.3%), were married. Furthermore, 270 (44.8%) had no formal education, and only 2.5% had higher educational status (Table 1).

**Table 1:** Baseline socio-demographic characteristics of HIV-positive adults on ART at Pawi General Hospital, northwest Ethiopia, Jan 2012–Dec 30, 2016

Variables	Frequency (n = 602)	Percent (%)
Age (years)		
15–28	189	31.4
29–34	160	26.6
35–44	170	28.2
> 45	83	13.8
Gender		
Female	338	56.2
Male	264	43.8
Marital status		
Single	112	18.6
Married	285	47.3
Divorced	146	24.3
Widowed	59	9.8
Educational status		
No formal education	270	44.8
Primary	222	36.9
Secondary	95	15.8
Higher	15	2.5
Occupation		
Daily labor	150	24.9
Farmer	200	33.2
Merchant	67	11.1
Government employee	48	8.0
Self-employee	42	7.0
Student	31	5.2
House wife	64	10.6

Nearly half, 313 (52%) of the patients were eligible for ART by WHO clinical stage and total lymphocyte count, while 289 (48%) were so by CD4 cell count criteria. The median baseline CD4 cell count was 217 (IQR: 119–341) cells/mm^3^. Regarding nutritional status, 255 (42.4%) of the participants were malnourished according to their body mass index (BMI). More than half, 325 (54.0%), of the participants were initiated into ART at WHO stage-III. Cotrimoxazole Preventive Therapy (CPT) and IPT were given to 418 (69.4%) and 340 (56.8%), respectively.

Over two-thirds, 410 (68.1%), of the patients started ART treatment with TDF–3TC–EFV and 36 (6%) changed to another regimen within the first line, and 11 (2%) switched to the second line. The majority of the participants, 444 (73.8%), were at working functional status. Over one-fifth, 133 (22.1%), had opportunistic infections, of whom 68 (11.3%) had Tuberculosis, 49 (8.1%) bacterial pneumonia and 16 (2.7%) other opportunistic infections (Table 2).

**Table 2:** Baseline clinical characteristics of HIV-positive adults on ART at Pawi General Hospital, northwest Ethiopia, Jan 1, 2012–Dec 30, 2016

Variables	Frequency (N = 602)	Percent (%)
IPT		
Not received	262	43.2
Received	340	56.8
CPT		
Not receiving	184	30.6
Receiving	418	69.4
Opportunistic infection		
No OI	469	77.9
Have OI	133	22.1
WHO clinical stage		
Stage I	55	9.1
Stage II	172	28.6
Stage III	325	54.0
Stage IV	50	8.3
ART regimen		
1c(AZT-3TC-NVP)	97	16.1
1d(AZT-3TC-EFV)	26	4.3
1e(TDF-3TC-EFV)	410	68.1
1f(TDF-3TC-NVP)	69	11.5
Functional status		
Working	444	73.8
Ambulatory	99	16.4
Bedridden	59	9.8
Adherence		
Good	585	97.2
Fair	12	2.0
Poor	5	0.8
Regimen change		
No	555	92.2
Yes		
First line	36	6.0
Second line	11	1.8
Nutritional status (BMI)		
Normal	347	57.6
Malnourished	255	42.4
CD4 cell count (cell/mm^3^)		
≤ 200	360	43.2
> 200	342	56.8

*AZT* zidovudine, *3TC* lamivudine, *NVP* nevirapine, *EFV* efavirenz, *TDF* tenofovier

The total follow-up time was 1175 adult-years observation with an incidence rate of 11.6 per 100 adult-years (95% CI 9.8–13.7) of LTFU. The subjects were followed for a minimum of three and a maximum of 60 months after initiation into ART with 18 months median follow-up time. The median ‘LTFU’ time of 15 months for male was shorter than 23 months for female patients. Nearly half of the subjects were LTFU within the first 6 months and around 73% before a year. The difference between two estimates of cumulative probability of LTFU (one minus Kaplan–Meier and cumulative incidence in the presence of death as a competing risk) is shown (Additional file 1). The additional file illustrated that the former overestimates the probability of experiencing LTFU at each follow-up time, except in the instance when there are no competing risks at the beginning of the follow up period.

The sub-hazard distribution proportionality assumption by schoenfeld residual global test was 0.4574, showed neither increasing nor decreasing pattern. As well, the Cox Snell residual plot almost overlay the Nelson–Aalen cumulative hazard (Additional file 2) showing the proportional sub-hazards assumption was satisfied.

Age, sex, CPT, WHO clinical stage, IPT, BMI, opportunistic infections, and functional status were fitted to the multivariable sub-distribution model. Accordingly, in the multivariable model adults in the over 45 years of age category lowered the sub-hazard ratio of LTFU by 56% (aSHR = 0.44; 95% CI 0.24–0.83) compared to those aged 15–28 years (Table 3). Adults who were on WHO clinical stage-IV increased the sub-hazard ratio of LTFU by 2.09 (aSHR = 2.09; 95% CI 1.19–3.67) times as compared to adults in stages-I and II. Being on IPT lowered the sub-distribution hazard ratio for LTFU by 89% (aSHR = 0.11; 95% CI 0.06–0.18) as compared to their counter parts.

**Table 3:** Multivariable competing risk regression analysis for predictors of LTFU among HIV-positive Adults at Pawi General Hospital, northwest Ethiopia, Jan 1, 2012–Dec 30, 2016

Variable	Survival status			cSHR [95% CI]	aSHR [95% CI]
	Lost (N = 136)	Death (N = 25)	Censored (N = 441)		
Age (years)					
15–28	49	6	134	1	1
29–34	34	9	117	0.72 [0.47–1.11]	0.76 (0.50–1.16)
35–45	41	5	124	0.86 [0.57–1.3]	0.85 (0.56–1.29)
> 45	12	5	66	0.47 (0.25–0.89)	0.44 (0.24–0.83)
Sex					
Female	63	12	262	1	1
Male	73	13	179	1.60 (1.14–2.23)	1.40 (0.99–1.99)
Nutritional status					
Normal	69	10	268	1	1
Malnourished	67	15	173	1.38 (0.99–1.93)	0.73 (0.52–1.02)
WHO clinical stage					
Stage (I/II)	36	5	186	1	1
Stage III	79	13	232	1.55 (1.05–2.29)	1.34 (0.92–1.95)
Stage IV	21	7	23	2.77 (1.62–4.76)	2.09 (1.19–3.67)
Functional status					
Asymptomatic	85	6	353	1	1
Symptomatic	51	19	88	1.63 (1.16–2.29)	1.03 (0.72–1.45)
Isoniazid					
Not received	115	20	125	1	1
Received	21	5	316	0.11 (0.07–0.17)	0.11 (0.07–0.18)
CPT					
Not received	46	12	126	1	1
Received	90	13	315	0.79 (0.56–1.12)	0.84 (0.59–1.20)
Opportunistic infection					
No	93	13	363	1	1
Yes	43	12	77	1.58 (1.12–2.23)	0.91 (0.63–1.30)

### Discussion

Several studies have shown that LTFU poses challenges to the successful implementation of ART programs in low resource settings [25]. This study aimed to assess the incidence of LTFU and its predictors among HIV positive adults after they were initiated into ART.

The incidence estimated in this study, 11.6 per 100 adult-years was consistent with 12.1–15.3 person-year reported in Latin and Caribbean countries, and 10.9 in South Africa [26, 27]. However, the incidence estimated was higher than those of studies in Zambia [9], and Aksum (northern-Ethiopia) [10]. The variation might be explained by differences in the study settings, transfer out, health seeking behavior, and late initiation of the community into ART at advanced WHO clinical stages-III and IV. The high rate of transfer out without prior information to their original health institution of initial registration necessary for proper recording may be another reason.

The proportion of LTFU of 22.6% seen in this study was lower than 36.6% reported in Cameroon [13] and 27.6% in South Africa [27]. It was, however, higher than the 15.5, 17.7, 15.6, 14.5, 19.6, and 21.7% detected in India, Sub-Saharan Africa, Nigeria, Hadiya (Southern-Ethiopia), Ethiopian public hospitals, and in Southern Nations Nationalities and Peoples (SNNP) Region (Ethiopia), respectively [11–13, 28–30]. The LTFU in our study turned out to be higher than those of the preceding reports because of travel costs patients had to cover to reach clinics. That means our study area is relatively under developed and sparsely populated with limited-infrastructure and resources which are directly or indirectly related to health service delivery and access.

The findings of the multivariable sub-distribution hazard regression revealed that, age category (15–28 years), not being on IPT prophylaxis, and WHO clinical stage-IV were independent predictors of LTFU. The finding implied that as age increased the probability of LTFU decreased. Patients aged above 45-years had lower risk of LTFU as compared to those aged 1528 years. This finding was consistent with those of studies conducted in Southern Nigeria, South Africa, Oromia and SNNP Region in Ethiopia [28, 31–34]. This could be due to immaturity in analytical thinking, and particular challenges associated with puberty. Fear of stigma and discrimination, as this younger age group is dependent on others, and independent members of this age group could be more mobile as compared to the older population [12]. This idea is also congruent with the qualitative findings indicating that the younger age groups who fear stigmatization are more prone to being LTFU from ART [35]. The finding is different from that of a study conducted in Nigeria [11] which showed younger groups were at a lower risk of LTFU.

HIV-positive adults in advanced clinical stage-IV increased the risk of LTFU by two-fold as compared to those in WHO clinical stage-I/II. This evidence was supported by studies conducted in South Africa, Uganda, India and Oromia (Ethiopia) [8, 12, 15, 36]. This is because more LTFU could be due to death and patients at advanced clinical stage are more likely to die because of side effects of ART within the first 6 months of ART initiation [37]. Beside severely ill patients might unable to revisit the service rendered. Another study in Nigeria indicated that half of the participants were in immunosuppressed advanced stages-III and IV [11].

Moreover, those who received IPT had a lower risk of LTFU as compared to those did not. This result is consistent with studies conducted in southern and northern Ethiopia, and Oromia (Ethiopia) [16, 31, 33]. The IPT prophylaxis recommended by national ART guidelines to prevent the occurrence of Tuberculosis co-infection, one of the causes of morbidity and mortality, might have been directly or indirectly imposed on LTFU.

### Conclusions

The incidence of LTFU was higher than in most studies in Ethiopia. HIV positive patients aged 15–28 years, WHO clinical stage-IV, and not being on IPT prophylaxes are at higher risk for LTFU. Those patients need support in terms of reminders, surveillance, and tracing mechanism to reduce LTFU.

## Limitations

Those persons who had no complete base line data were not included. Predictors like viral load, hemoglobin test, and clinical factors were not included as they were not well documented on patient cards. Thus, this finding should be interpreted with this limitation in mind.

## Additional files

**Additional file 1.** Kaplan-Meier failure curve and cumulative incidence function for Lost to follow up.

**Additional file 2.** Cox Snell residual plot for overall fitness of the model.

## References

[1] WHO. US/HIV AIDS report. 2015.

[2] UNAIDS. HIV/AIDS report. 2014.

[3] HAPCO. Federal Democratic Republic of Ethiopia, HIV/AIDS Prevention and Control Office. 2014.

[4] Cohen MS, Chen YQ, McCauley M, Gamble T, Hosseinipour MC, Kumarasamy N (2011). Prevention of HIV-1 infection with early antiretroviral therapy. N Engl J Med.

[5] World Health Organization (2017). HIV/AIDS fact sheet.

[6] Estill J, Tweya H, Egger M, Wandeler G, Feldacker C, Johnson LF (2014). Tracing of patients lost to follow-up and HIV transmission: mathematical modelling study based on two large ART programmes in Malawi. J Acquir Immune Defic Syndr (1999).

[7] Taiwo B (2009). Understanding transmitted HIV resistance through the experience in the USA. Int J Infect Dis.

[8] Mberi MN, Kuonza LR, Dube NM, Nattey C, Manda S, Summers R (2015). Determinants of loss to follow-up in patients on antiretroviral treatment, South Africa, 2004–2012: a cohort study. BMC Health Serv Res.

[9] Li MS, Musonda P, Gartland M, Mulenga PL, Mwango A, Stringer JS (2013). Predictors of patient attrition according to different definitions for loss to follow-up: a comparative analysis from Lusaka, Zambia. J Acquir Immune Defic Syndr (1999).

[10] Tadesse K, Fisiha H (2014). Predictors of loss to follow up of patients enrolled on antiretroviral therapy: a retrospective cohort study. J AIDS Clin Res.

[11] Eguzo KN, Lawal AK, Eseigbe CE, Umezurike CC (2014). Determinants of mortality among adult HIV-infected patients on antiretroviral therapy in a rural hospital in Southeastern Nigeria: a 5-year cohort study. AIDS Res Treat.

[12] Alvarez-Uria G, Naik PK, Pakam R, Midde M (2013). Factors associated with attrition, mortality, and loss to follow up after antiretroviral therapy initiation: data from an HIV cohort study in India. Glob Health Action.

[13] Bekolo CE, Webster J, Batenganya M, Sume GE, Kollo B (2013). Trends in mortality and loss to follow-up in HIV care at the Nkongsamba Regional hospital, Cameroon. BMC Res Notes.

[14] Palombi L, Marazzi MC, Guidotti G, Germano P, Buonomo E, Scarcella P (2009). Incidence and predictors of death, retention, and switch to second-line regimens in antiretroviral-treated patients in sub-Saharan African Sites with comprehensive monitoring availability. Clin Infect Dis.

[15] Assefa Y, Lynen L, Kloos H, Hill P, Rasschaert F, Hailemariam D (2015). Brief report: long-term Outcomes and their determinants in patients on antiretroviral treatment in Ethiopia, 2005/6–2011/12: a retrospective cohort study. JAIDS J Acquir Immune Defic Syndr.

[16] Dessalegn M, Tsadik M, Lemma H (2015). Predictors of lost to follow up to antiretroviral therapy in primary public hospital of Wukro, Tigray, Ethiopia: a case control study. J AIDS HIV Res.

[17] Koller MT, Raatz H, Steyerberg EW, Wolbers M (2012). Competing risks and the clinical community: irrelevance or ignorance?. Stat Med.

[18] Fine JP, Gray RJ (1999). A proportional hazards model for the subdistribution of a competing risk. J Am Stat Assoc.

[19] Lau B, Cole SR, Gange SJ (2009). Competing risk regression models for epidemiologic data. Am J Epidemiol.

[20] Cox D (1972). Regression models and life tables. J Roy Stat Soc Ser B..

[21] Satagopan J, Ben-Porat L, Berwick M, Robson M, Kutler D, Auerbach A (2004). A note on competing risks in survival data analysis. Br J Cancer.

[22] Chi BH, Yiannoutsos CT, Westfall AO, Newman JE, Zhou J, Cesar C (2011). Universal definition of loss to follow-up in HIV treatment programs: a statistical analysis of 111 facilities in Africa, Asia, and Latin America. PLoS Med.

[23] Bakoyannis G, Touloumi G (2012). Practical methods for competing risks data: a review. Stat Methods Med Res.

[24] Gooley TA, Leisenring W, Crowley J, Storer BE (1999). Estimation of failure probabilities in the presence of competing risks: new representations of old estimators. Stat Med.

[25] Rosen S, Fox MP, Gill CJ (2007). Patient retention in antiretroviral therapy programs in sub-Saharan Africa: a systematic review. PLoS Med.

[26] Carriquiry G, Fink V, Koethe JR, Giganti MJ, Jayathilake K, Blevins M (2015). Mortality and loss to follow-up among HIV-infected persons on long-term antiretroviral therapy in Latin America and the Caribbean. J Int AIDS Soc.

[27] Dalal RP, MacPhail C, Mqhayi M, Wing J, Feldman C, Chersich MF (2008). Characteristics and outcomes of adult patients lost to follow-up at an antiretroviral treatment clinic in Johannesburg, South Africa. JAIDS J Acquir Immune Defic Syndr.

[28] Teshome W, Belayneh M, Moges M, Mekonnen E, Endrias M, Ayele S (2015). Do loss to follow-up and death rates from ART care vary across primary health care facilities and hospitals in south Ethiopia? A retrospective follow-up study. HIV/AIDS (Auckland, NZ).

[29] Wilhelmson S, Reepalu A, Tolera Balcha T, Jarso G, Björkman P (2016). Retention in care among HIV-positive patients initiating second-line antiretroviral therapy: a retrospective study from an Ethiopian public hospital clinic. Glob Health Action.

[30] Ayele W, Mulugeta A, Desta A, Rabito FA (2015). Treatment outcomes and their determinants in HIV patients on anti-retroviral treatment program in selected health facilities of Kembata and Hadiya zones, Southern Nations, Nationalities and Peoples Region, Ethiopia. BMC Public Health.

[31] Shargie EB, Lindtjørn B (2007). Determinants of treatment adherence among smear-positive pulmonary tuberculosis patients in Southern Ethiopia. PLoS Med.

[32] Wang B, Losina E, Stark R, Munro A, Walensky RP, Wilke M (2011). Loss to follow-up in a community clinic in South Africa: roles of gender, pregnancy and CD4 count. SAMJ S Afr Med J.

[33] Megerso A, Garoma S, Tolosa Eticha TW, Daba S, Tarekegn M, Habtamu Z (2016). Predictors of loss to follow-up in antiretroviral treatment for adult patients in the Oromia region, Ethiopia. HIV/AIDS (Auckland, NZ).

[34] Meloni ST, Chang C, Chaplin B, Rawizza H, Jolayemi O, Banigbe B (2014). Time-dependent predictors of loss to follow-up in a large HIV treatment cohort in Nigeria. Open Forum Infect Dis.

[35] Wubshet M, Berhane Y, Worku A, Kebede Y (2013). Death and seeking alternative therapy largely accounted for lost to follow-up of patients on ART in northwest Ethiopia: a community tracking survey. PLOS One.

[36] Nakiwogga-Muwanga A, Alamo-Talisuna S, Musaazi J, Kambugu A, Ssekawungu P, Katabira E (2014). Inadequate monitoring in advanced stages of disease with lack of supportive counseling increases attrition among patients on antiretroviral treatment at a large urban clinic in Uganda. J Int Assoc Provid AIDS Care (JIAPAC).

[37] Bhatta L, Klouman E, Deuba K (2013). Survival on antiretroviral treatment among adult HIV-infected patients in Nepal: a retrospective cohort study in far-western region. BMC Infect Dis.

