# Supplementary figures and images for: Incidence and predictors of loss to follow up among HIV-infected adults at Pawi General Hospital, northwest Ethiopia: competing risk regression model
Source: BMC Res Notes. 2018 May 10;11:287. doi: 10.1186/s13104-018-3407-5 (PMC5946498; doi:10.1186/s13104-018-3407-5)

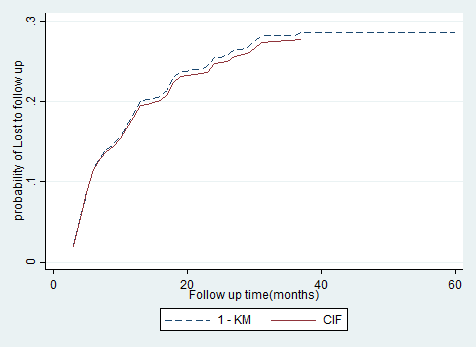

Supplement: Supplementary file 1 — Additional file 1. Kaplan-Meier failure curve and cumulative incidence function for Lost to follow up. [file 13104_2018_3407_MOESM1_ESM.tif]

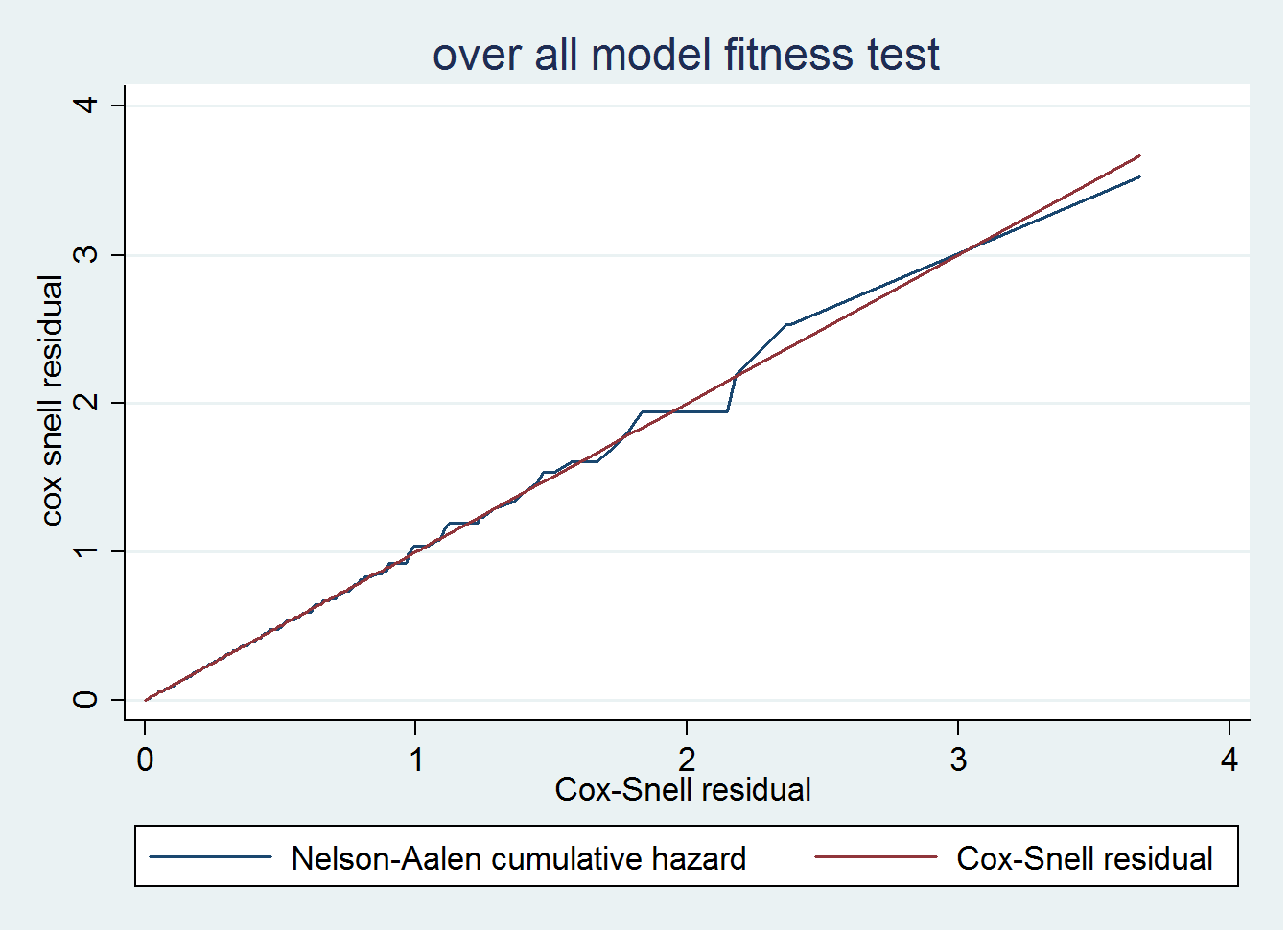

Supplement: Supplementary file 2 — Additional file 2. Cox Snell residual plot for overall fitness of the model. [file 13104_2018_3407_MOESM2_ESM.tif]
